# Polysomnographic Predictors of Treatment Response to Cognitive Behavioral Therapy for Insomnia in Participants With Co-morbid Insomnia and Sleep Apnea: Secondary Analysis of a Randomized Controlled Trial

**DOI:** 10.3389/fpsyg.2021.676763

**Published:** 2021-05-04

**Authors:** Alexander Sweetman, Bastien Lechat, Peter G. Catcheside, Simon Smith, Nick A. Antic, Amanda O’Grady, Nicola Dunn, R. Doug McEvoy, Leon Lack

**Affiliations:** ^1^The Adelaide Institute for Sleep Health and Flinders Health and Medical Research Institute: Sleep Health, Flinders University, Adelaide, SA, Australia; ^2^College of Medicine and Public Health, Flinders University, Adelaide, SA, Australia; ^3^College of Science and Engineering, Flinders University, Adelaide, SA, Australia; ^4^Institute for Social Science Research, The University of Queensland, Brisbane, QLD, Australia; ^5^Sleep Health Service, Repatriation General Hospital and Respiratory and Sleep Services, Southern Adelaide Local Health Network, Adelaide, SA, Australia; ^6^Thoracic Program, The Prince Charles Hospital, Chermside, QLD, Australia; ^7^College of Education Psychology and Social Work, Flinders University, Adelaide, SA, Australia

**Keywords:** COMISA, chronic insomnia, sleep disordered breathing, obstructive sleep apnea, Polysomnogram, precision medicine, CBTi, qEEG

## Abstract

**Objective:**

Co-morbid insomnia and sleep apnea (COMISA) is a common and debilitating condition that is more difficult to treat compared to insomnia or sleep apnea-alone. Emerging evidence suggests that cognitive behavioral therapy for insomnia (CBTi) is effective in patients with COMISA, however, those with more severe sleep apnea and evidence of greater objective sleep disturbance may be less responsive to CBTi. Polysomnographic sleep study data has been used to predict treatment response to CBTi in patients with insomnia-alone, but not in patients with COMISA. We used randomized controlled trial data to investigate polysomnographic predictors of insomnia improvement following CBTi, versus control in participants with COMISA.

**Methods:**

One hundred and forty five participants with insomnia (ICSD-3) and sleep apnea [apnea-hypopnea index (AHI) ≥ 15] were randomized to CBTi (*n* = 72) or no-treatment control (*n* = 73). Mixed models were used to investigate the effect of pre-treatment AHI, sleep duration, and other traditional (AASM sleep macrostructure), and novel [quantitative electroencephalography (qEEG)] polysomnographic predictors of between-group changes in Insomnia Severity Index (ISI) scores from pre-treatment to post-treatment.

**Results:**

Compared to control, CBTi was associated with greater ISI improvement among participants with; higher AHI (interaction *p* = 0.011), less wake after sleep onset (interaction *p* = 0.045), and less N3 sleep (interaction *p* = 0.005). No quantitative electroencephalographic, or other traditional polysomnographic variables predicted between-group ISI change (all *p* > 0.09).

**Discussion:**

Among participants with COMISA, higher OSA severity predicted a greater treatment-response to CBTi, versus control. People with COMISA should be treated with CBTi, which is effective even in the presence of severe OSA and objective sleep disturbance.

## Introduction

Chronic insomnia and obstructive sleep apnea (OSA) are the two most common sleep disorders and frequently co-occur within the same patient (The American Academy of Sleep Medicine, 2014; Sweetman et al., 2017a). Chronic insomnia is characterized by frequent difficulties initiating sleep, maintaining sleep, and/or early morning awakenings from sleep, and associated daytime functional impairments, which persist for at least 3 months (The American Academy of Sleep Medicine, 2014). OSA is characterized by frequent narrowing (hypopnea) or collapse (apnea) of the upper airway during sleep, resulting in reduced oxygen saturation, frequent cortical arousals, and daytime sleepiness and fatigue. The clinical index of OSA is the apnea-hypopnea index (AHI) which reflects the average number of respiratory events per hour of sleep. Moderate and severe OSA are commonly defined by an AHI of 15–29, and ≥30 events/h of sleep, respectively. Insomnia and OSA both increase risk of mental health problems (Peppard et al., 2006; Baglioni et al., 2011), and result in substantial societal costs through reduced productivity and quality of life, and high healthcare utilization (Ozminkowski et al., 2007; Wickwire et al., 2016; Natsky et al., 2020).

Co-morbid insomnia and sleep apnea (COMISA) is a common and debilitating condition (Luyster et al., 2010; Sweetman et al., 2017a, 2019a). For example, approximately 30–50% of patients with OSA report clinically significant insomnia symptoms, while 30–40% of patients with insomnia fulfill diagnostic criteria for OSA when assessed via overnight polysomnography (Zhang et al., 2019; Sweetman et al., 2019a). Compared to patients with insomnia-alone, or OSA-alone, patients with COMISA experience greater impairment to sleep (Bianchi et al., 2013), mood (Lang et al., 2017), daytime functioning (Krakow et al., 2001; Smith et al., 2004; Sweetman et al., 2017b), and quality of life (Sweetman et al., 2017a, 2019a; Tasbakan et al., 2018).

Despite the high prevalence and morbidity associated with COMISA, the most effective treatment approach is unknown. Continuous positive airway pressure (CPAP) therapy is the first-line treatment for moderate and severe OSA (Epstein et al., 2009). However, patients with COMISA show lower initial acceptance and long-term use of CPAP, compared to patients with OSA alone (Suraiya and Lavie, 2006; Wickwire et al., 2010; Pieh et al., 2012; Sweetman et al., 2017a; Philip et al., 2018). On the other hand, cognitive behavioral therapy for insomnia (CBTi) is the recommended “first-line” treatment for insomnia (Schutte-Rodin et al., 2008; Qaseem et al., 2016). Although CBTi leads to a significant overall reduction in insomnia symptoms among patients with COMISA (Fung et al., 2014; Sweetman et al., 2017b, 2019b; Alessi et al., 2020; Ong et al., 2020), the efficacy of CBTi is variable between patients, with up to 65% of COMISA patients failing to achieve remission of insomnia by post-CBTi (Sweetman et al., 2017b). Therefore, it is important to identify which patients with COMISA are likely to respond to CBTi, to guide tailored treatment approaches (Sweetman et al., 2017a).

Polysomnographic sleep study data have previously been used to predict response to CBTi in patients with insomnia (Bathgate et al., 2017; Miller et al., 2018; Kalmbach et al., 2020). For example, studies have reported that shorter sleep duration (Bathgate et al., 2017), lower objective sleep efficiency (Kalmbach et al., 2020; percentage of time in bed spent asleep), shorter sleep onset latency (Troxel et al., 2013), specific quantitative electroencephalography (qEEG) parameters (Krystal and Edinger, 2010), and co-morbid OSA (Sweetman et al., 2017b) are associated with a reduced response to CBTi.

In the context of COMISA, it is possible that patients who have more severe OSA (higher AHI), worse sleep macrostructure (less sleep time, sleep efficiency), or lighter and more fragmented sleep (more light sleep, higher arousal index, and modified sleep microstructure) before treatment may be less responsive to CBTi. This information is critical to guide the most appropriate treatment approach for patients with COMISA. For example, if patients with insomnia symptoms and severe OSA are minimally responsive to CBTi, it may be possible to expedite CPAP therapy to manage the OSA, before commencing CBTi. Alternatively, if CBTi is found to be effective in the presence of more severe OSA, it may be possible to treat such patients with CBTi to improve insomnia and potentially increase use of CPAP therapy (Sweetman et al., 2019b; Alessi et al., 2020; Ong et al., 2020). Therefore, we aimed to use randomized controlled trial (RCT) data to investigate the effect of pre-treatment polysomnographic sleep and respiratory data on insomnia improvements following CBTi, versus control in participants with COMISA.

## Materials and Methods

### Study Design

This study used data from a previously reported RCT investigating the effect of a 4-session CBTi program, versus no-treatment control, on subsequent acceptance and use of CPAP therapy in participants with COMISA (Sweetman et al., 2019b). As reported previously (Sweetman et al., 2019b), the CBTi group reported a greater average reduction in the ISI from pre-treatment to post-treatment, compared to the control group. This exploratory study aimed to investigate whether any polysomnographic data before treatment predicted change in insomnia symptoms from pre-treatment to post-treatment, in the CBTi group, versus the control group. Mixed model analyses were used to investigate the interaction effect between pre-treatment polysomnographic predictors, group (CBTi, control) and time (pre-treatment, post-treatment) on self-reported insomnia symptoms. No participants were using CPAP therapy during this study phase. Participants with previous CPAP use were not excluded, provided that CPAP had not been used for at least 3 months before the pre-treatment assessment. 10 of 72 participants in the CBTi group and 8 of 73 participants in the control group reported previous CPAP use (Fisher’s exact *p* = 0.624).

### Participant Screening and Recruitment

**FIGURE 1:**
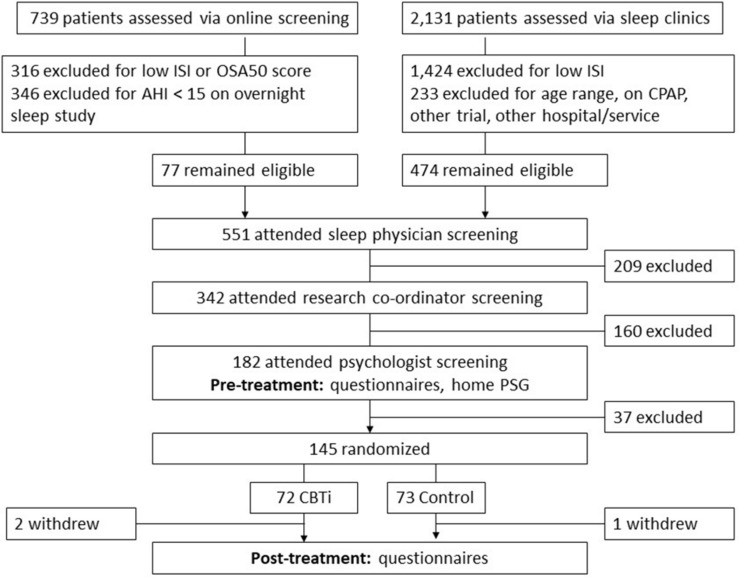
Flow diagram indicating participant screening, randomization and follow-up. ISI, insomnia severity index; OSA50, obstructive sleep apnea 50 questionnaire; AHI, apnea–hypopnea index/h; and CPAP, continuous positive airway pressure.

Participant screening and recruitment have been described previously (Sweetman et al., 2019b), and are illustrated in Figure 1. Briefly, two Australian outpatient sleep clinics screened 2,870 patients, of who 145 people with untreated insomnia [Psychologist diagnosis; ICSD-3 criteria (The American Academy of Sleep Medicine, 2014)] and OSA (Sleep physician diagnosis; AHI ≥ 15 according to full overnight sleep study) were randomized between November 2013 and April 2016.

### Cognitive Behavioral Therapy for Insomnia and Control

Following completion of pre-treatment questionnaires, sleep diaries, and overnight polysomnography, participants were randomly allocated to a CBTi group or to a control group. A manualized CBTi program including four 45-min sessions was administered by registered or provisional psychologists trained in administration of this CBTi intervention.

Session 1 included sleep education (two-process model of sleep regulation), and bedtime restriction therapy. Session 2 included sleep misperception feedback (Polysomnography versus sleep diary parameters). Session 3 included information about insomnia, hyper-arousal, and cognitive therapy. Session 4 was used to review the previous weeks of therapy and for relapse prevention. Participants completed sleep diaries and an Epworth Sleepiness Scale during each session. CBTi did not include any information about OSA or CPAP therapy.

Adequate treatment fidelity and effectiveness of CBTi, versus control has been reported previously (Sweetman et al., 2019b). Participants randomized to the control condition received no treatment between pre-treatment and post-treatment.

### Polysomnographic Predictors

Participants completed a single home-based diagnostic polysomnographic study before randomization (Somté, Compumedics, Australia). Participants attended sleep laboratories on the evening of sleep studies to be set up with recording equipment by trained technicians. Polysomnography studies included electrode placement to record two electroencephalographic channels (C3–M2, C4–M1), two chin-electromyogram channels, two electrooculogram channels, two leg-electromyogram channels, a nasal cannula to record nasal pressure, a oro-nasal thermistor, a finger pulse oximeter, and chest and abdominal bands to record respiratory effort. As participants were free to choose their time in bed parameters in their home environment, there was no systematic restriction of time in bed on the night of the polysomnography study. Studies were scored by trained technicians according to AASM 2007 “alternate” criteria (Iber et al., 2007; Ruehland et al., 2009). Three main categories of polysomnographic predictors were investigated. Firstly, sleep apnea severity predictors included; AHI, arousal index, and nadir oxygen saturation. Secondly, we examined traditional AASM sleep parameters and sleep architecture predictors including; total sleep time, sleep efficiency, sleep onset latency, wake after sleep onset, and total sleep time in each stage (N1–3, REM).

Finally, we examined qEEG predictors, including absolute and relative non-rapid eye movement sleep and rapid eye movement sleep beta, alpha, theta, and delta power, and N2 K-complex density. Briefly, absolute power of the beta, alpha, theta, and delta power was calculated in 5-s non-overlapping EEG segments using multi-taper based (Prerau et al., 2017) fast Fourier transform as reported previously (Scott et al., 2020). The mean absolute power in NREM epochs was then calculated for each frequency band. Epochs with an absolute maximum amplitude of 400 μv were considered noisy. Our choice of rejection threshold for noisy epochs is inevitably somewhat arbitrary, but was chosen to exceed the largest slow wave EEG events of interest during sleep; which have a negative maximum amplitude of around 200 to 300 μv (Massimini et al., 2004). We have previously reproduced more complex noise rejection algorithms (D’Rozario et al., 2015). However, the difference between the current threshold (maximum amplitude of 400 μv) and these more complex algorithms was very small (around 4 min over the full night). Thus, we opted for the simpler threshold (max 400 μv) for this study since it is more easily implemented for others to reproduce. K-complexes were scored according to previously published method (Lechat et al., 2020) and K-complex density was defined as the number of K-complexes occurrence divided by the time spent in N2 sleep. Although no frontal electrodes were included in our PSG montage, this K-complex density algorithm has previously been validated based on C3 electrode placement that was used in the current study.

All polysomnographic variables were retained as continuous predictors to preserve information that is otherwise lost by splitting continuous data into two dichotomous groups (Altman and Royston, 2006). However, as a sensitivity analysis, and to inform clinical utility of the effect of OSA severity on treatment-response, OSA severity was also investigated by dividing participants into two groups reflecting standard clinical cut-offs for moderate (AHI < 30) versus severe OSA (AHI ≥ 30). Similarly, polysomnographic sleep duration was also investigated by dividing participants into those with short (<6 h), versus normal sleep duration (≥6 h) according to previously established categories (Bathgate et al., 2017).

### Insomnia Severity Index

The Insomnia Severity Index (ISI) was the primary outcome measure. The ISI is a 7-item self-report measure of insomnia severity which has been used extensively in insomnia-treatment research (Bastien et al., 2001). Scores range from 0 to 28, with higher scores indicating more severe insomnia symptoms. A reduction of ≥8 points was used to define “responders” (Sweetman et al., 2019b), and a post-treatment threshold of ≥15 was used to indicate “clinically significant insomnia” (Bastien et al., 2001; Morin et al., 2011; Gagnon et al., 2013).

### Statistical Analyses

Data were analyzed with IBM SPSS (Version 25) on an intention-to-treat basis. Mixed models were used to examine interaction effects between pre-treatment polysomnographic predictors, group (CBTi, control) and time (pre-treatment, post-treatment) on the ISI. Binary logistic regression was used to investigate the effect of pre-treatment polysomnographic predictors on rates of “clinically significant insomnia” by post-treatment. A small amount of missing data and patterns of missing data have been reported previously (two participants in the CBTi group and one participant in the control group withdrew before post-treatment; Figure 1; Sweetman et al., 2019b). Descriptive statistics are reported with standard deviations (pre-treatment data), and 95% confidence intervals (*CI*s; change data) and an alpha significance level of <0.05 was used.

Data were extracted for qEEG analysis from both electroencephalogram channels (C^3^–M^2^, C^4^–M^1^). These were initially retained as independent predictors (e.g., EEG1 beta power, EEG2 beta power, etc.). As no significant predictors were observed when analyzing qEEG power of separate channels, the average qEEG power of both channels was calculated and reported in the analyses below.

## Results

### Pre-treatment Information and Overall Effect of CBTi versus Control

Participants included 145 people with COMISA (Age *M* = 58.2, *sd* = 9.9; 55% male). There were no between-group differences in any socio-demographic, questionnaire, or polysomnographic variables at pre-treatment, including the proportion of participants with severe OSA (AHI ≥ 30), or the proportion of participants with normal sleep duration (sleep duration ≥6 h; see Table 1).

**TABLE 1:** Average (sd) pre-treatment socio-demographic and clinical characteristics between groups.

	CBTi	Control	*p*
	*M* (SD)	*M* (SD)	
Age, years	59.1 (9.9)	57.3 (9.9)	0.279
Sex, no. (%) male	40 (55.6)	40 (54.8)	0.927^∧^
Body mass index	34.5 (6.3)	36.2 (6.5)	0.107
Insomnia severity index	18.5 (5.4)	17.9 (4.7)	0.497
**One-week sleep diary**			
Total sleep time (min)	346.2 (74.5)	350.6 (80.8)	0.59
Sleep onset latency (min)	56.6 (49.8)	49.6 (34.9)	0.274
Wake after sleep onset (min)	94.5 (60.4)	99.3 (72.3)	0.486
Sleep efficiency (%)	66.7 (12.4)	68.0 (14.5)	0.573
**Polysomnographic data**			
Total sleep time (min)	375.2 (86.1)	358.4 (87.1)	0.245
Total sleep time ≥6 h, *n* (%)	43 (60.6)	37 (50.7)	0.233^∧^
Sleep onset latency (min)	32.5 (63.7)	29.2 (33.2)	0.697
Wake after sleep onset (min)	95.7 (63.3)	82.1 (49.5)	0.153
Sleep efficiency (%)	74.3 (14.1)	75.0 (11.9)	0.757
AHI	33.2 (19.8)	35.8 (23.9)	0.471
Severe OSA (AHI ≥ 30), *n* (%)	34 (48.6)	34 (36.6)	0.811^∧^
Arousal index	32.7 (17.0)	35.6 (22.2)	0.377
N1 (min)	84.2 (49.9)	82.5 (58.0)	0.855
N2 (min)	179.9 (56.0)	164.7 (62.7)	0.129
N3 (min)	48.9 (37.9)	54.6 (38.2)	0.368
REM (min)	62.3 (29.4)	56.5 (30.7)	0.25

*All data are displayed as means and standard deviations, unless otherwise specified. ∧Chi^2^ test. AHI, apnea/hypopnea index/h; ISI, Insomnia severity index; OSA, obstructive sleep apnea; and REM, rapid eye movement.*

As reported previously (Sweetman et al., 2019b), the CBTi group experienced a greater ISI reduction from pre-treatment (*M* = 18.5, ±95%*CI* = 1.3) to post-treatment (*M* = 12.2, ±95%*CI* = 1.4) compared to the control group (pre-treatment *M* = 18.0, ±95%*CI* = 1.3; post-treatment *M* = 16.6, ±95%*CI* = 1.4; and interaction *p* < 0.001). This pattern of results was similar when investigating each polysomnography predictor of treatment response (below). Therefore, the two-way (group by time) analyses are not reported for each model.

### Polysomnographic Predictors of Response to Cognitive Behavioral Therapy for Insomnia

#### Sleep Apnea Severity

**FIGURE 2:**
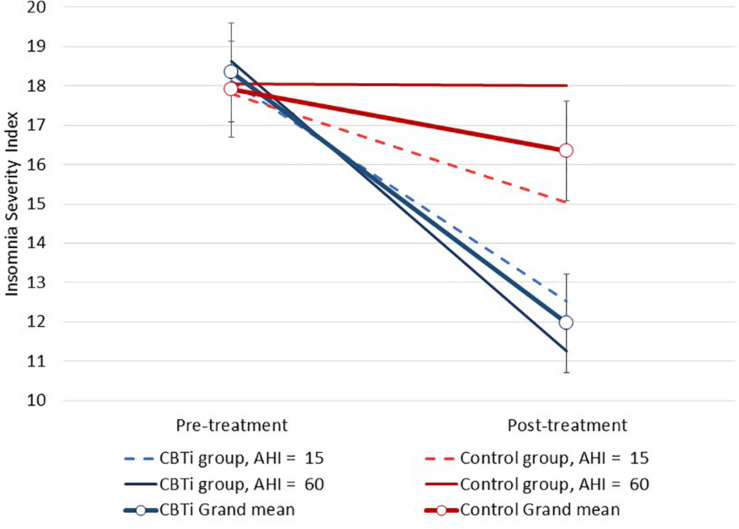
Modeled data illustrating the effect of pre-treatment AHI, group (CBTi, control), and time (pre-treatment, post-treatment) on the Insomnia Severity Index (95%CI). Pre-treatment AHI was retained as a continuous predictor and data are plotted for pre-treatment AHI values of 15 and 60 for each group according to model intercept, slopes and interaction effects. Compared to control, higher pre-treatment AHI was associated with greater insomnia severity reduction during CBTi.

Interaction effects between continuous polysomnography variables, group and time on the ISI are displayed in Table 2. There was a three-way interaction effect between pre-treatment AHI (continuous), group (CBTi, versus control), and time (pre-treatment, versus post-treatment) on the ISI [Figure 2; *F*(1,134) = 6.7, interaction *p* = 0.011]. Higher pre-treatment AHI was associated a greater reduction of insomnia severity in the CBTi group, but a smaller reduction of insomnia severity in the control group from pre-treatment to post-treatment. Put differently, among participants with more severe OSA (e.g., AHI = 60, solid darker lines; Figure 2), control participants experienced very little ISI reduction, while CBTi participants experienced a large ISI reduction by post-treatment. However, among participants with less severe OSA (e.g., AHI = 15, dashed lines; Figure 2), there was less difference in reduction of ISI between the CBTi and control group by post-treatment. As pre-treatment AHI was retained as a continuous predictor in this model, these AHI values were chosen for illustrative purposes (Figure 2).

**TABLE 2:** Effect of continuous polysomnography parameters on ISI change during CBTi, versus control, represented as beta coefficient (±95%*CI*).

Polysomnography parameter		All participants		CBTi group-only	
		**Three-way interaction**		**Two-way interaction**	
**Parameter**	**Increment**	***p*-value**	**Beta coefficient**	***p*-value**	**Beta coefficient**
Total sleep time	(per 30-min)	0.098	−0.24 (0.28)	**0.047**	**−0.45 (0.44)**
Sleep onset latency	(per 30-min)	0.488	0.46 (1.30)	**0.012**	**0.76 (0.59)**
Wake after sleep onset	(per 30-min)	**0.045**	**0.93 (0.91)**	0.435	−0.02 (0.62)
Sleep efficiency	(per 10%)	0.881	0.10 (1.32)	0.174	−1.91 (2.77)
AHI	(per 15-units)	**0.011**	**1.50 (1.14)**	0.229	0.07 (1.96)
Arousal index	(per 15-units)	0.121	1.03 (2.60)	0.626	0.28 (2.31)
N1 sleep	(per 30-min)	0.840	0.10 (0.93)	0.290	−0.42 (0.78)
N2 sleep	(per 30-min)	0.811	−0.10 (0.86)	0.665	−0.15 (0.70)
N3 sleep	(per 30-min)	**0.005**	**−1.89 (1.31)**	0.231	−0.62 (1.02)
REM sleep	(per 30-min)	0.056	−1.65 (1.69)	0.110	−1.08 (1.33)

*All polysomnography parameters are adjusted to reflect common clinical threshold. This means that beta coefficients represent amount of ISI change per 30-min increase of sleep time parameters (e.g., total sleep time, sleep onset latency, wake after sleep onset, N1-3, and REM sleep), 10% increase in sleep efficiency, and per 15-unit increments of respiratory indices [Apnea hypopnoea index/h sleep (AHI) and arousal index]. Beta coefficient = ISI change per one-increment change in polysomnography parameter. Significant associations are indicated with bold font. Three-way interactions = group (CBTi, control) × time (Pre-treatment, post-treatment) × polysomnography parameter (continuous). E.g., each one-increment change in the apnea hypopnea index/h sleep (AHI increase of 15-units) was associated with a 1.5-point greater ISI reduction by post-treatment in the CBTi, versus the control group. Two-way interaction = time (pre-treatment, post-treatment) × polysomnography parameter. E.g., each one-increment increase in total sleep time (increase of 30-min) was associated with a 0.45-point greater reduction in ISI by post-treatment.*

This effect was supported in a sensitivity analysis of the interaction between AHI severity level (<30, versus ≥30), group, and time on the ISI (interaction *p* = 0.028; Supplementary Figure 1). Among participants with severe OSA, those in the control group showed no reduction of ISI by post-treatment (*M* reduction = 0.25, ±95%*CI* = 1.7, *p* = 0.77, and Cohen’s *d* = 0.05), while those in the CBTi group showed a large ISI reduction (*M* reduction = 7.0, ±95%*CI* = 1.7, *p* < 0.001, and Cohen’s *d* = 1.4). Among participants with moderate OSA, significant ISI reduction by post-treatment was observed in both the control (*M* reduction = 2.6, ±95%*CI* = 1.7, *p* = 0.002, and Cohen’s *d* = 0.5) and CBTi groups (*M* reduction = 5.7, ±95%*CI* = 1.6, *p* < 0.001, and Cohen’s *d* = 1.1). There was no effect of pre-treatment arousal index [continuous; *F*(1,133) = 2.4, interaction *p* = 0.121], or nadir oxygen saturation [continuous; *F*(1,133) <0.1, interaction *p* = 0.943] on between-group change in the ISI by post-treatment.

When examining only participants in the CBTi group, there were no interaction effects of pre-treatment AHI [*F*(1,67) = 1.5, interaction *p* = 0.229], arousal index [*F*(1,67) = 0.2, interaction *p* = 0.626], or nadir oxygen saturation [*F*(1,67) = 0.1, interaction *p* = 0.754] (all retained as continuous predictors) on change in the ISI by post-treatment.

### Sleep Duration and Sleep Efficiency

**FIGURE 3:**
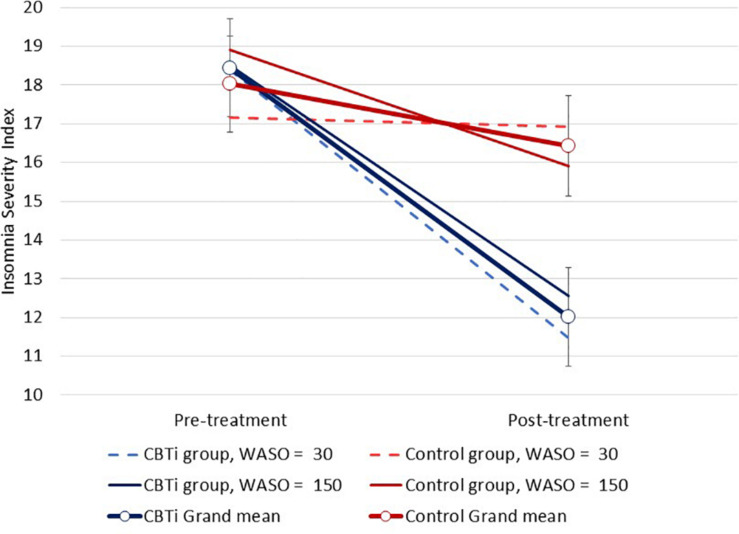
Modeled data illustrating the effect of pre-treatment wake after sleep onset (WASO; min), group (CBTi, control), and time (pre-treatment, post-treatment) on the Insomnia Severity Index (CI). Pre-treatment WASO was retained as a continuous predictor and data are plotted according to intercept, slopes and interaction effects. Compared to control, lower pre-treatment WASO predicted greater insomnia severity reduction in the CBTi group.

**FIGURE 4:**
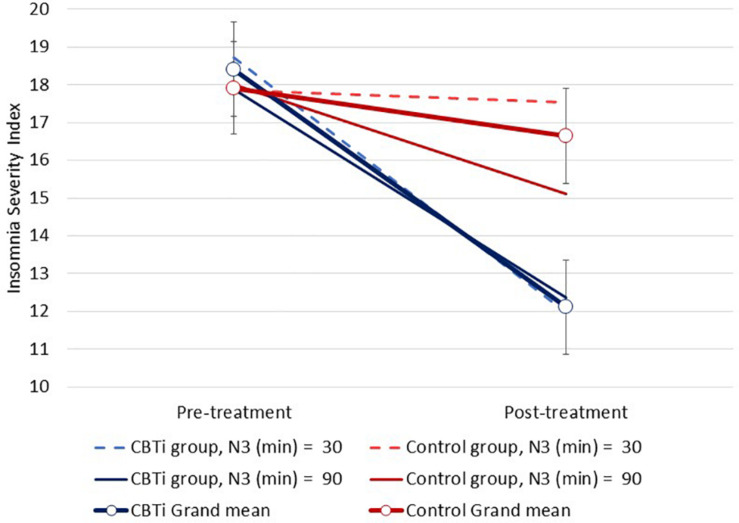
Modeled data illustrating the effect of pre-treatment N3 sleep (min), group (CBTi, control), and time (pre-treatment, post-treatment) on the Insomnia Severity Index (CI). Pre-treatment N3 sleep was retained as a continuous predictor and data are plotted according to intercept, slopes and interaction effects. Compared to control, less N3 sleep time predicted greater insomnia severity reduction in the CBTi group.

**FIGURE 5:**
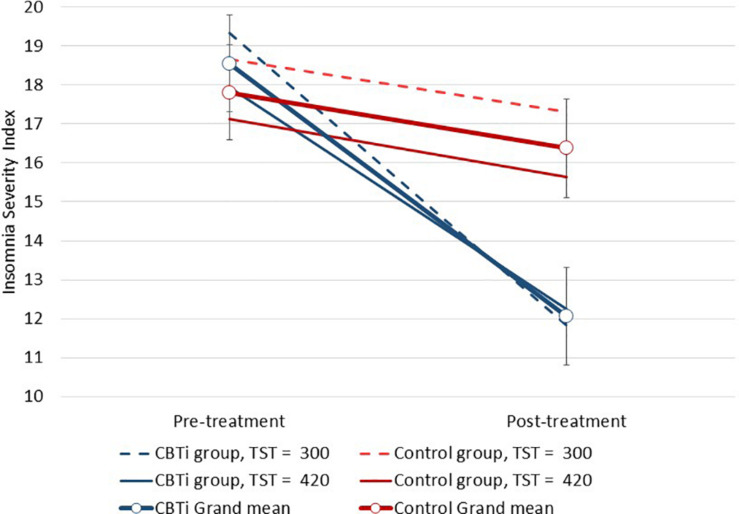
Modeled data illustrating the effect of pre-treatment total sleep time, group (CBTi, control), and time (pre-treatment, post-treatment) on the Insomnia Severity Index (95%CI; not statistically significant). Pre-treatment sleep time was retained as a continuous predictor and data are plotted according to intercept, slopes and interaction effects.

Several three-way interactions between group, time, and polysomnography sleep parameters (retained as continuous variables) on ISI were performed. Compared to control, greater ISI reduction in the CBTi group was predicted by less objective wake after sleep onset [*F*(1,134) = 4.1, interaction *p* = 0.045; Figure 3], and less time spent in N3 sleep [*F*(1,135) = 8.2, interaction *p* = 0.005; Figure 4]. There was no effect of pre-treatment polysomnographic total sleep time [*F*(1,133) = 2.8, interaction *p* = 0.098; Figure 5], sleep efficiency [*F*(1,137) <0.1, interaction *p* = 0.881], sleep onset latency [*F*(1,145) = 0.5, interaction *p* = 0.488], or time spent in N1 [*F*(1,133) <0.1, interaction *p* = 0.840], N2 [*F*(1,134) <0.1, interaction *p* = 0.811], or REM sleep [*F*(1,136) = 3.7, interaction *p* = 0.056; see Supplementary Figure 2] on reduction of ISI scores during treatment between the CBTi versus control group.

When total sleep time was dichotomized into short (<6 h) versus normal (≥6 h) sleep duration, there was no three-way interaction effect of sleep duration group, intervention-group and time on the ISI (*p* = 0.341). Indeed, among participants in the control group, there was no significant ISI reduction by post-treatment in the short (*M* reduction = 1.38, 95%CI = 1.7, *p* = 0.11, and Cohen’s *d* = 0.3) or normal sleep duration groups (*M* reduction = 1.49, 95%CI = 1.7, *p* = 0.08, and Cohen’s *d* = 0.3). Among participants in the CBTi group, there was a significant ISI reduction by post-treatment in both the short (*M* reduction = 7.30, 95%CI = 1.9, *p* < 0.001, and Cohen’s *d* = 1.4) and normal sleep duration groups (*M* reduction = 5.76, 95%CI = 1.7, *p* < 0.001, and Cohen’s *d* = 1.1).

When analyses were restricted to only participants in the CBTi group, there was a significant interaction effect between polysomnographic total sleep duration (continuous) and time [*F*(1,67) = 4.1, interaction *p* = 0.047; see Figure 5], and sleep onset latency and time (interaction *p* = 0.012) on the ISI. Shorter sleep duration (see slopes for CBTi group in Figure 5) and longer sleep onset latency before treatment (i.e., greater sleep impairment) were associated with a greater reduction of ISI scores from pre-treatment to post-treatment. When sleep duration was dichotomized into short (<6 h) versus normal sleep duration (≥6 h), the interaction between sleep duration and time on ISI among participants in the CBTi group was no longer significant (*p* = 0.251). There was no effect of continuous polysomnographic wake after sleep onset (interaction *p* = 0.435), sleep efficiency (interaction *p* = 0.174), or time spent in any sleep stage (N1–3, REM; all interaction *p* ≥ 0.11) on reduction of ISI scores during treatment in the CBTi group.

#### Sleep Fragmentation and Quantitative Electroencephalography

Finally, qEEG predictors of treatment-response to CBTi were also investigated in a series of exploratory analyses. There was no effect of pre-treatment absolute or ratio (relative to power across other bands) non-rapid eye movement sleep beta, alpha, theta, or delta power, or N2 K-complex density on reduction of ISI scores between groups (all retained as continuous predictors; all *p* > 0.09). Similarly, there was no effect of rapid eye movement sleep beta, alpha, theta, or delta power on reduction of ISI scores between groups (all retained as continuous predictors; all *p* > 0.30).

When analyses were restricted to participants in the CBTi group, there were no significant interaction effects between any non-rapid eye movement or rapid eye movement sleep absolute qEEG predictors and time on ISI scores (all retained as continuous predictors; all *p* ≥ 0.10).

### Responder Analyses

Among participants in the CBTi group, binary logistic regression was used to investigate the association of pre-treatment polysomnographic data with rates of insomnia response (≥8 point reduction by post-treatment), and “clinically significant insomnia” (ISI ≥ 15) by post-treatment.

Participants in the CBTi group reported lower rates of “clinically significant insomnia” at post-treatment (34.8%), compared to participants in the control group (65.2%; χ^2^ = 12.5, *p* < 0.001). There was a statistically significant, albeit small, association between pre-treatment non-rapid eye movement sleep absolute beta power and “clinically significant insomnia” classification at post-treatment (exponentiated B = 0.99; *p* = 0.048). No other pre-treatment polysomnographic variables were associated with rates of clinically significant insomnia by post-treatment (all *p* ≥ 0.13). Among those in the CBTi group, there was no difference in rates of clinically significant insomnia between participants with moderate (40%) versus severe OSA (29.4%) by post-treatment (χ^2^ = 0.85, *p* = 0.11).

Participants in the CBTi group were more likely to show a positive insomnia response from pre-treatment to post-treatment (ISI reduction ≥ 8; 46.4%), compared to participants in the control group (6.1%; χ^2^ = 28.0, *p* < 0.001). There was a statistically significant, albeit small, association between pre-treatment sleep efficiency and rates of insomnia response in the CBTi group (exponentiated B = 0.96, *p* = 0.046). No other polysomnography variables before treatment were associated with rates of insomnia response in the CBTi group (all *p* > 0.05). Among those in the CBTi group, there was no difference in rates of “insomnia response” between participants with moderate (37.1%) versus severe OSA (55.9%) by post-treatment (χ^2^ = 2.4, *p* = 0.12).

## Discussion

The main finding of this study was that among participants with COMISA, we found that more severe OSA before treatment was associated with a greater reduction of insomnia severity during CBTi, versus control. Polysomnographic sleep duration, other measures of sleep fragmentation (arousal index, time spent in light sleep), and qEEG sleep parameters before treatment did not predict a reduced response to CBTi, versus control. This suggests that CBTi is effective in the presence of severe OSA, and in participants who commence treatment with greater objective sleep disturbance.

Although it was hypothesized that participants with COMISA who have more severe OSA and evidence of greater objective sleep disturbance may be less responsive to CBTi, these data indicate that CBTi is an effective treatment among such participants. Theoretically, among patients with more severe OSA, clinicians may conceptualize the insomnia as a secondary manifestation of the untreated OSA. Indeed, respiratory events and sleep fragmentation may result in awakenings that precipitate insomnia symptoms in some patients with OSA. Previous studies have also demonstrated that CPAP therapy improves insomnia symptoms among some COMISA patients, including those with more severe OSA (Björnsdóttir et al., 2013; Glidewell et al., 2014). However, the present data indicate that greater OSA severity does not predict a reduced response to CBTi among people with both disorders.

In fact, participants with more severe OSA a reported greater reduction of insomnia severity following CBTi, versus control. This three-way interaction effect was largely due to variability in changes in insomnia severity over time in the control group, while the CBTi group showed more homogenous improvement of insomnia across different OSA severity levels (Figure 2). Among control participants, this variable effect of pre-treatment AHI on reduction of insomnia may result from a natural reduction of insomnia severity over time or gradual regression to the mean among participants with less severe OSA, while participants with more severe OSA experienced more stable insomnia severity between pre-treatment and post-treatment. Given that un-treated insomnia symptoms showed little natural variation over time among participants with more severe OSA, it is recommended that clinicians consider referring such patients for CBTi to improve insomnia and potentially increase subsequent CPAP use (Sweetman et al.,2019a,b).

Previous studies have investigated the effect of short objective sleep duration (i.e., total sleep time < 6 h) and objective sleep efficiency on treatment-response to CBTi in patients with insomnia (Bathgate et al., 2017; Galbiati et al., 2019; Kalmbach et al., 2020). It has been postulated that insomnia patients with short objective sleep duration may experience an insomnia phenotype characterized by underlying biological mechanisms, rather than psychological/behavioral mechanisms that may be less responsive to CBTi (Vgontzas et al., 2013). In the current COMISA sample, polysomnographic sleep duration and sleep efficiency did not predict response to CBTi, versus control. When analyses were restricted to only participants in the CBTi group, short sleep duration and longer sleep onset latency predicted greater ISI reduction during CBTi (e.g., see CBTi group in Figure 5). Although these findings potentially indicate a greater response to CBTi among participants with greater objective sleep impairment before treatment, these interaction effects were no longer statistically significant when comparing with changes across time in the control group. It is likely that the underlying mechanisms proposed to underpin the short sleep duration phenotype differ between people with insomnia-alone and those with COMISA (Bianchi et al., 2013; Vgontzas et al., 2013). For example, among COMISA participants, it is possible that sleep duration is partially dependent on frequent respiratory events causing arousals, and awakenings, in addition to physiological arousal processes. The “short sleep duration” insomnia phenotype requires further investigation in the context of COMISA.

We observed that participants with less objective wake after sleep onset, and less time spent in N3 sleep experienced a greater reduction of insomnia severity following CBTi, versus control. Similar to the three-way interaction of AHI, group and time on insomnia severity, these interaction effects also largely resulted from variability in changes in insomnia severity in the control group, but homogenous reduction in the CBTi group. Finally, we conducted exploratory analyses to investigate if any novel qEEG parameters predicted treatment-response to CBTi, but observed no notable or statistically significant predictors.

## Limitations

Although this study had a number of strengths including the use of standardized measures and interventions, and only a small amount of missing data (Sweetman et al., 2019b), there are important limitations that warrant consideration. Firstly, this was a secondary analysis of existing RCT data, which was not specifically powered to identify polysomnographic predictors of treatment-response to CBTi. However, as previous studies with smaller sample sizes have detected statistically significant effects of objective sleep duration (Bathgate et al., 2017), sleep efficiency (Kalmbach et al., 2020), sleep onset latency (Troxel et al., 2013), and qEEG predictors (Krystal and Edinger, 2010) on response to CBTi in patients with insomnia, it is unlikely that our exploratory study was under-powered to detect an effect of these specific polysomnographic predictors on treatment-response.

Secondly, only a single night of polysomnographic data was collected before treatment, which may have been impacted by “first night effects,” or night-to-night variability in objective sleep and respiratory parameters. Given the high costs associated with polysomnography, it may be possible for future studies to utilize novel and less expensive measures of objective sleep such as wearable wrist-worn or electroencephalography devices (e.g., actigraphy, portable EEG monitoring devices) capable of measuring sleep over several consecutive nights to produce more stable estimates of key sleep parameters (Arnal et al., 2020).

Finally, the generalizability of this study is restricted to participants with at least moderate OSA (AHI ≥ 15). As this study did not include insomnia participants with no OSA or mild OSA, it was not possible to determine whether the presence/absence of sleep apnea (or mild severity OSA) is predictive of treatment response to CBTi. A previous study investigating the effectiveness of CBTi between patients with insomnia-alone and COMISA (mild-severe OSA) reported that although both groups showed significant improvement of insomnia during CBTi, those with COMISA reported a slightly smaller response to CBTi by 3-month follow-up (Sweetman et al., 2017b). Instead, the present study targeted an important clinical question related to the most effective treatment approach in patients with both chronic insomnia and moderate/severe OSA.

## Conclusion

Emerging evidence suggests that CBTi may be an effective (Sweetman et al., 2019b; Alessi et al., 2020; Ong et al., 2020) and safe (Sweetman et al., 2020) overall treatment for COMISA, however, it is important to identify which patients are most responsive to CBTi, and which patients are less responsive to CBTi and require other initial treatments (e.g., CPAP). Contrary to our hypothesis, this study found that higher OSA severity before treatment was associated with a greater insomnia improvement following CBTi, compared with control. Furthermore, objective short sleep duration, and qEEG parameters did not predict a reduced response to CBTi, versus control in participants with COMISA. Compared to control, CBTi was associated with greater ISI improvement among participants with less objective wake after sleep onset, and less N3 sleep before treatment. This suggests that people with COMISA should be referred for CBTi, regardless of OSA severity or objective sleep disturbance before treatment.

## Data Availability Statement

The datasets presented in this article are not readily available because participants did not provide informed consent for sharing of trial data.

## Ethics Statement

The studies involving human participants were reviewed and approved by the Southern Adelaide Clinical Human Research Ethics Committee (Southern Adelaide Local Health Network, Adelaide, Australia), the Human Research Ethics Committee (The Prince Charles Hospital, Brisbane, Australia), the Queensland University of Technology Human Research Ethics Committee, and the External Request Evaluation Committee (Services Australia). The patients/participants provided their written informed consent to participate in this study.

## Author Contributions

AS: study design, data collection, management, and manuscript writing and analysis. BL: extraction of qEEG data and manuscript drafting. PC: study design, management, and manuscript drafting. SS: study design, management, and manuscript drafting. NA: study design and management. AO’G: data collection and management. ND: data collection and management. RDMc: study design, management, and manuscript drafting. LL: study design, management, and manuscript drafting. All authors have approved this manuscript.

## Conflict of Interest

RDMc reports research funding support from Philips Respironics and research equipment donations from ResMed and Air Liquide. PC received salary support via an Australian Research Council Future Fellowship (FT120100510), reports research funding support from Philips Respironics via the CRC for Alertness, Safety and Productivity, and equipment support from Philips Respironics and Air Liquide. LL has received funding support from Re-timer (Re-timer Pty Ltd, Adelaide, Australia). The remaining authors declare that the research was conducted in the absence of any commercial or financial relationships that could be construed as a potential conflict of interest.
