# Supplementary material for: Polysomnographic Predictors of Treatment Response to Cognitive Behavioral Therapy for Insomnia in Participants With Co-morbid Insomnia and Sleep Apnea: Secondary Analysis of a Randomized Controlled Trial
Source: Front Psychol. 2021 May 4;12:676763. doi: 10.3389/fpsyg.2021.676763 (PMC8129160; doi:10.3389/fpsyg.2021.676763)
Supplement: Supplementary file 1 [file Data_Sheet_1.docx]

**Supplementary Materials**

Sweetman et al. Polysomnographic predictors of treatment response to cognitive behavioral therapy for insomnia in participants with co-morbid insomnia and sleep apnea: Secondary analysis of a randomized controlled trial.

A sensitivity analysis was performed to investigate the effect of sleep apnea severity group (AHI <30, versus ≥30), intervention group (cognitive behavioral therapy for insomnia [CBTi], versus control), and time (pre-treatment, versus post-treatment) on the Insomnia Severity Index (ISI; Figure S1). A significant three-way interaction (*p* = 0.028) confirmed the results of the primary analysis (in which AHI was retained as a continuous predictor). Among participants with severe sleep apnoea, those in the control group showed no change in ISI from pre-treatment to post-treatment, while those in the CBTi group showed a large ISI reduction. Among participants with moderate sleep apnoea, significant ISI reduction was observed in both to control and CBTi groups.

Figure S1. Effect of moderate, versus severe sleep apnea, intervention-group and time on the Insomnia Severity Index.

An interaction between rapid eye movement sleep (min), intervention-group and time on the Insomnia Severity Index approached statistical significance (interaction *p* = 0.056; Figure S2). Among participants with a greater amount of rapid eye movement sleep, there was little difference in ISI reduction between the CBTi and control group by post-treatment. Among participants with less rapid eye movement sleep, those in the CBTi group showed a pattern of larger ISI reduction compared to those in the control group. These data are plotted below for interest, however as this interaction effect did not reach statistical significance, these data should be interpreted with caution.

Figure S2. Effect of rapid eye movement (REM) sleep (min), intervention group and time on the Insomnia Severity Index.
